# Improved identification and enrolment into care of HIV-exposed and -infected infants and children following a community health worker intervention in Lilongwe, Malawi

**DOI:** 10.7448/IAS.18.1.19305

**Published:** 2015-01-07

**Authors:** Saeed Ahmed, Maria H Kim, Amanda C Dave, Rachael Sabelli, Kondwani Kanjelo, Geoffrey A Preidis, Thomas P Giordano, Elizabeth Chiao, Mina Hosseinipour, Peter N Kazembe, Frank Chimbwandira, Elaine J Abrams

**Affiliations:** 1 Baylor International Pediatric AIDS Initiative at Texas Children's Hospital, Baylor College of Medicine, Houston, TX, USA; 2 Baylor College of Medicine-Abbott Fund Children's Clinical Center of Excellence, Lilongwe, Malawi;; 3 Department of Medicine, Baylor College of Medicine, Houston, TX, USA; 4 UNC Project, Lilongwe, Malawi; 5 Department of Medicine, University of North Carolina, Chapel Hill, NC, USA; 6 HIV Unit, Malawi Ministry of Health, Lilongwe, Malawi; 7 ICAP, Mailman School of Public Health and College of Physicians & Surgeons, Columbia University, New York, NY, USA

**Keywords:** case finding, linkage to care, children, HIV, paediatrics, Africa, community health workers, HIV-exposed infants

## Abstract

**Background:**

Early identification and entry into care is critical to reducing morbidity and mortality in children with HIV. The objective of this report is to describe the impact of the Tingathe programme, which utilizes community health workers (CHWs) to improve identification and enrolment into care of HIV-exposed and -infected infants and children.

**Methods:**

Three programme phases are described. During the first phase, Mentorship Only (MO) (March 2007–February 2008) on-site clinical mentorship on paediatric HIV care was provided. In the second phase, Tingathe-Basic (March 2008–February 2009), CHWs provided HIV testing and counselling to improve case finding of HIV-exposed and -infected children. In the final phase, Tingathe-PMTCT (prevention of mother-to-child transmission) (March 2009–February 2011), CHWs were also assigned to HIV-positive pregnant women to improve mother-infant retention in care. We reviewed routinely collected programme data from HIV testing registers, patient mastercards and clinic attendance registers from March 2005 to March 2011.

**Results:**

During MO, 42 children (38 HIV-infected and 4 HIV-exposed) were active in care. During Tingathe-Basic, 238 HIV-infected children (HIC) were newly enrolled, a six-fold increase in rate of enrolment from 3.2 to 19.8 per month. The number of HIV-exposed infants (HEI) increased from 4 to 118. During Tingathe-PMTCT, 526 HIC were newly enrolled over 24 months, at a rate of 21.9 patients per month. There was also a seven-fold increase in the average number of exposed infants enrolled per month (9.5–70 patients per month), resulting in 1667 enrolled with a younger median age at enrolment (5.2 vs. 2.5 months; *p*<0.001).

During the Tingathe-Basic and Tingathe-PMTCT periods, CHWs conducted 44,388 rapid HIV tests, 7658 (17.3%) in children aged 18 months to 15 years; 351 (4.6%) tested HIV-positive. Over this time, 1781 HEI were enrolled, with 102 (5.7%) found HIV-infected by positive PCR. Additional HIC entered care through various mechanisms (including positive linkage by CHWs and transfer-ins) such that by February 2011, a total of 866 HIC were receiving care, a 23-fold increase from 2008.

**Conclusions:**

A multipronged approach utilizing CHWs to conduct HIV testing, link HIC into care and provide support to PMTCT mothers can dramatically improve the identification and enrolment into care of HIV-exposed and -infected children.

## Introduction

Within the past 10 years, great progress has been made in the prevention of mother-to-child transmission (PMTCT) of HIV infection, early infant diagnosis (EID) of HIV and care and treatment of HIV-exposed infants (HEI) and HIV-infected children (HIC). As currently available PMTCT strategies capable of reducing transmission rates to less than 5% are possible even in breastfeeding populations, UNAIDS has declared a goal of “virtual elimination” by 2015 [1]. High-burden countries are steadily expanding access to EID utilizing DNA PCR testing [2–4]. With the increasing availability of co-trimoxazole prophylaxis (CPT) and antiretroviral therapy (ART), outcomes for HIC have dramatically improved, not only in developed countries but also in resource-limited settings [5–7]. Over 600,000 children are currently receiving ART globally [8].

Despite these advances, considerable challenges remain, with the most significant being access to care. HIV-infected infants suffer from rapid immunologic deterioration, disease progression and high mortality without early ART initiation [9,10], yet only 20% of HEI in resource-limited settings receive an HIV test [11]. There are an estimated 3.3 million children infected with HIV, with 1.9 million needing ART [8]. Strengthening case finding and prompt entry into care is essential to ensure these children benefit from life-saving care and antiretroviral treatment services [12].

Improving identification and enrolment into care is also critical for older children and adolescents. Up to one third of infected infants have slower progressing disease and may survive into their teenage years without treatment [13]. There are few venues or opportunities for identifying older children, with the majority not presenting until they are clinically ill, increasing mortality risk and compromising treatment outcomes [14,15].

Community-based HIV testing has been proposed as one strategy to improve paediatric case finding [12]. Results from pilot programmes suggest that community-based testing is acceptable and feasible, can reduce disparities in access to testing services and is cost-effective for increasing HIV testing among previously unreached populations [16–19]. Home-based HIV testing, compared to voluntary testing and counselling and provider-initiated testing and counselling, can identify symptomatic and asymptomatic patients earlier in their disease course [20]. Task shifting with use of community health workers (CHWs) and lay counsellors for community-based testing is especially suited to settings with limited human resource capacity [21–28]. However, the majority of programmes have focused on adults [18,20,29–34].

The Baylor College of Medicine Children's Foundation Malawi (BCM-CFM) is the largest provider of paediatric HIV care in Malawi, with over 20,000 patients enrolled at the Clinical Center of Excellence and satellite clinics. In 2007, BCM-CFM partnered with the Malawi Ministry of Health (MOH) and Lilongwe District Health Office to initiate an outreach programme, called Tingathe (meaning “yes we can” in the local Chichewa language), which utilizes CHWs to improve uptake and utilization of PMTCT, EID and paediatric HIV care services. The objective of this report is to summarize these efforts and examine their impact on identification and enrolment into care of HIV-exposed and -infected infants and children.

## Methods

### Setting and patient population

This report describes Tingathe activities from 2007 to 2011 at three MOH health centres in Lilongwe, Malawi: Area 25, Area 18 and Kawale. These clinics serve a combined catchment area of close to 500,000 people, with 12,000 women attending antenatal clinic annually, and an antenatal HIV prevalence of 10.1% [23,35]. During this time, there was no nationally established programme to systematically provide pre-ART care for HIV-positive persons or -exposed infants.

### Programme phases

**Figure 1:**
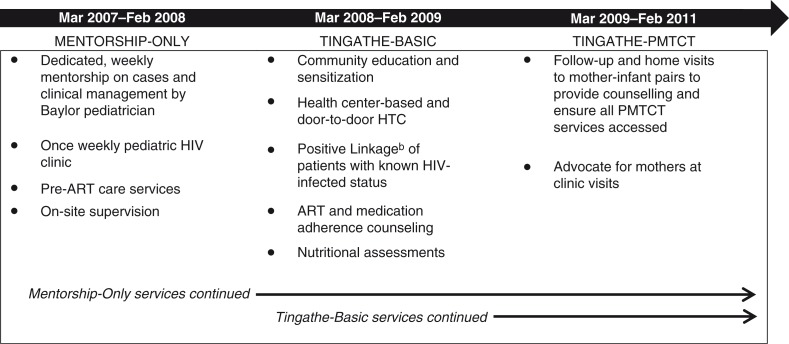
Summary of Tingathe intervention – services offered^a^. ART,=antiretroviral treatment; PMTCT=prevention of mother-to-child transmission; HTC=HIV testing and counselling. ^a^Clinical space, HIV test kits and supplies, antiretroviral and other medications were provided by the Malawi MOH. ^b^Positive linkage refers to linking children with known HIV-infected status to clinical care services.

Three programme phases are described within this report (Figure 1).

### Mentorship Only (MO): March 2007–February 2008

In March 2007, BCM-CFM started a programme of clinical mentorship at these sites. Paediatricians trained in the United States provided on-site mentorship of government clinical care providers. Mentorship was provided during a dedicated, once weekly paediatric HIV clinic where both HEI and HIC were seen and regular pre-ART care was offered including clinical staging and CD4 measurements. All HIV care including rapid HIV testing, HIV DNA PCR testing via dried blood spot (DBS), WHO staging [36], CD4 testing, CPT and ART was provided at no cost by the MOH.

### Tingathe-Basic (T-Basic): March 2008–February 2009

In March 2008, BCM-CFM initiated the Tingathe Community Outreach Programme. CHWs were recruited and trained in community sensitization and education, health-centre- and community-based HIV testing and counselling (HTC), as well as active case finding of patients. Details of the Tingathe intervention, including how CHWs were recruited, selected, trained, supervised and remunerated are summarized in Table 1 [23,37].

**Table 1:** Tingathe community health worker characteristics, training, responsibilities and supervision

**Requirements**	Completion of primary schooling Residence in catchment area Ability to ride a bicycle Ability to read and write in English HIV-infected or HIV-affected
**Recruitment**	Group interviews by programme leadership followed by individual interviews
**Training**	Four-week MOH HTC training Two-week specialized Baylor paediatric HIV and PMTCT HIV training, plus two-week on-site orientation Half-day, quarterly refresher training by Baylor paediatricians
**Training topics**	Basics of HIV/AIDS PMTCT: what are the steps and how to promote utilization of services Caring for the exposed infant (importance of EID and CPT) Diagnosing HIV infection Positive linkage of patients with known HIV-infected status Nutrition: exclusive breastfeeding; malnutrition screening Children with HIV: identification, care and treatment ART and adherence counselling Reducing stigma and discrimination Counselling and community mobilization and education skills Conducting patient home visit
**Number**	Five to six per site during **T-Basic** Eight to ten per site during **T-PMTCT** One SS per site
**Responsibilities**	Health centre navigation Health talks and education at the health centre HTC at the health centre and community Patient home visits and adherence support
**Payment and incentives**	$50–$100 per month per CHW; bicycle for transportation; mobile phone airtime; bags for supplies and materials
**Patient load**	50–100 clients at one time
**Supervision**	SS confirmation of competency before allowing home visits Supervised weekly by SS and monthly by programme coordinator Biannual performance evaluations SS conducted unscheduled patient visits without CHW to ensure patient satisfaction

MOH=Ministry of Health; HTC=HIV testing and counselling; PMTCT=prevention of mother-to-child transmission; EID=early infant diagnosis; CPT=co-trimoxazole prophylaxis; CHW=community health worker; SS=site supervisor.

Positive linkage refers to linking children with known HIV-infected status to clinical care services.

All CHWs were trained in HTC, utilizing rapid antibody-based testing. CHWs conducted both health-centre-based and home-based testing. When the programme was initiated, testing was restricted to the health centre. As counsellors became more proficient in HTC, they began to solicit referrals for home-based testing of untested family members of current ART patients. After several months of referral-based testing, door-to-door testing was initiated. The majority of testing was conducted in either the home or health centre; with some additional testing conducted in other venues including community sensitization events, orphanages and youth services facilities (grouped under the category “other” in the analysis).

All of the children tested by Tingathe CHWs had an indication for testing such as a parent being infected, being an orphan or a specific request from a guardian for testing. Adults were usually tested first, and only if the parent tested positive were the children tested. If the mother was uninfected and there were no other indications for testing, the children were not tested. Children older than 18 months were tested with antibody-based rapid tests using finger-pricks. Exposed infants under the age of 18 months were tested using DBS for HIV DNA PCR testing through the national EID programme. Consent/assent for testing was obtained according to MOH guidelines [38].

CHWs also attempted to identify children with known HIV-infected status who were not in clinical care, a process we referred to as positive linkage. Positive linkage was based on the premise that many guardians knew that their children were infected but were not aware that services were available for infected children. Strategies for positive linkage included asking adult ART patients if they had HIC and checking the status (by asking guardians and checking health records) of children at potentially high-prevalence settings such as tuberculosis and outpatient malnutrition clinics, as well as orphanages located within the community [12]. If they were already known to be HIV-infected but not in clinical care, Tingathe CHWs supported their linkage into clinical care.

CHWs were not only responsible for identifying HIC but also enrolling them into clinical care and providing adherence support. When children and adolescents with HIV infection were identified, they were enrolled into the programme and assigned a CHW. The CHW would follow the child at home and in clinic to ensure that he or she enrolled into care, maintained good adherence and received all available services including clinical and immunologic staging, CPT and ART, if appropriate. CHWs utilized one-page patient mastercards to document and track clinic appointments, home visits, services utilized, new diagnoses and test results.

Clinical mentorship by Baylor clinicians continued during this time period.

### Tingathe-PMTCT (T-PMTCT): March 2009–February 2011

During the T-Basic phase, it was noted that effective linkage of HEI into care was still suboptimal. Therefore, in March 2009, a PMTCT component was added that assigned CHWs to HIV-positive pregnant women at antenatal care. CHWs supported mother-infant pairs to access all available PMTCT services including CD4 testing, initiation of ART for the mother, provision of ARV prophylaxis for the child, testing and diagnosis of HEI, counselling on infant feeding and enrolment of infected infants into care. They followed the women at their homes and at the health centres, from initial diagnosis up until cessation of breastfeeding and final testing of infants, with either negative diagnosis and discharge or successful enrolment of infected infants into care. HIC were followed regularly to ensure that they were receiving appropriate services. Full details of this PMTCT intervention are described elsewhere [23,37].

All the activities of the MO and T-Basic phases continued during this time period.

### Study design and statistical analysis

We reviewed routinely collected programme data from patient mastercards, clinic rosters, PMTCT registers and HIV testing registers from March 2007 to March 2011. All data were de-identified prior to analysis. The overall objective was to assess the impact of Tingathe activities on identification and enrolment. Four main outcomes were examined:

1. Identification of HIV-positive persons via HIV testing efforts. Data from HIV testing registers were analyzed.
2. New enrolment of HEI and HIC into care. Clinic enrolment numbers and rates were calculated from clinic rosters. For clarity and to prevent double counting, all patients were categorized as either exposed (HEI) or infected (HIC) by their status at enrolment. Even if a HEI was later determined to be HIV-infected, he or she was not included in the HIC group in this analysis of new enrolment.
3. Changes in patient characteristics. Patient level data (age, gender, PCR information) from mastercards and clinic registers were analyzed. Clinical and immunologic staging were determined using WHO classification guidelines [36].
4. Total enrolment and mechanism of identification of HIC. For this analysis, in addition to all patients who were HIC at enrolment, those who were HEI at enrolment, but later found to be HIV-infected with a positive PCR, were also included. Patient mastercards and registers provided information on mechanism of identification.

Aggregate data were reported as mean with standard deviation or median with interquartile range (IQR) based on normality. Continuous non-parametric variables were evaluated with the Mann–Whitney U test. For categorical parameters, data were reported as raw value and percentage of the respective group. Analyses were performed using IBM SPSS Statistics (version 19; SPSS, Inc., Chicago, IL, USA).

### Ethical review

Both the Malawi National Health Sciences Research Committee and the Baylor College of Medicine institutional review board approved this study.

## Results

### HIV testing using antibody-based rapid tests

During the three years of the T-Basic and T-PMTCT phases, CHWs conducted 44,388 rapid HIV tests, 7658 (17.3%) in children aged 18 months to 15 years (Table 2). Testing strategy, testing numbers and number as positive identified did not change during the two programme phases. Of the 7658 children tested, 351 (4.6%) had a positive rapid HIV antibody test. Health-centre-based testing demonstrated a significantly higher prevalence of HIC than home-based testing (12.4% vs. 3.0%; *p*<0.001). However, four times more children were tested through the home-based strategy, resulting in roughly equivalent numbers of HIC being identified (163 vs. 159).

**Table 2:** HIV testing results for children aged 18 months^a^ to 15 years (March 2008–February 2011)

Characteristics		Tested	Positive^b^
Total, *N* (%)		7658 (100)	351 (4.6)
Gender, *n* (%)	Male	3411 (44.5)	148 (4.3)
	Female	4247 (55.5)	203 (4.8)
Prior testing	Tested before	818 (10.7)	53 (6.5)
status, *n* (%)	Never tested before	6840 (89.3)	298 (4.4)
Age, *n* (%)	1.5–5	2664 (34.8)	140 (5.3)
	>5–15	4994 (65.2)	211 (4.2)
Testing location,	Health centre	1310 (17.1)	163 (12.4)
*n* (%)	Home	5372 (70.1)	159 (3.0)
	Other^c^	976 (12.7)	29 (3.0)

a Children under 18 months could not be tested using antibody-based rapid tests as per Malawi guidelines;

b denominator for positive percentage is the number tested for that characteristic;

c other venues including community sensitization events, orphanages and youth services facilities.

Table 2 describes characteristics of children aged 18 months to 15 years that received HTC through the Tingathe programme.

### New enrolment of HEI and HIC

During the MO phase, there were 42 children active in care at the three centres. Of these, 38 were HIV-infected and four were HIV-exposed.

During the T-Basic phase, 238 HIC were enrolled at the three clinics, a six-fold increase in rate of enrolment for HIC from 3.2 to 19.8 per month from pre- to post-intervention periods. The number of HEI increased from 4 to 118.

During the T-PMTCT phase, 526 HIC were enrolled over 24 months, with a slight increase in the rate of enrolment from 19.8 to 21.9 patients per month. However, the addition of PMTCT activities resulted in a greater than seven-fold increase in the average number of HEI enrolled per month (9.5**–**70 patients per month) and in younger median age at enrolment (5.2 [IQR 2.8–9.8] to 2.5 [IQR 1.6–4.8] months; *p*<0.001). A total of 1667 HEI enrolled during the two-year period.

Overall, during the three years following the introduction of the Tingathe intervention, a total of 2545 new paediatric patients, 764 HIC and 1781 HEI, were newly enrolled at the three participating clinics.

**Figure 2:**
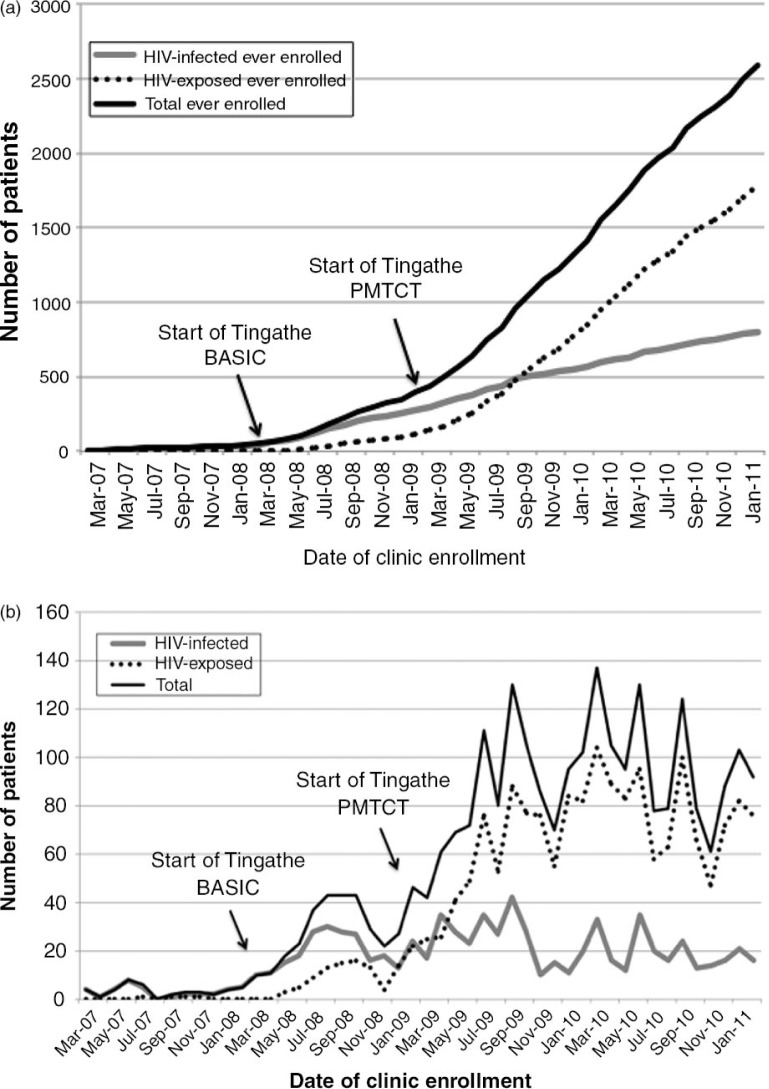
(a) New enrolment and (b) rate of new enrolment of HIV-exposed infants (HEI) and HIV-infected children (HIC).

Figure 2a depicts total enrolment and Figure 2b depicts rates of enrolment as described above.

### Characteristics of HEI at enrolment

Characteristics of the patients who were HIV-exposed at enrolment are presented in Table 3.

**Table 3:** Characteristics of HIV-exposed infants (HEI) and early infant diagnosis (EID) testing results

Characteristic	Mentorship Only (Mar 07–Feb 08) *N*=4	Tingathe-Basic (Mar 08–Feb 09) *N*=114	Tingathe-PMTCT (Mar 09–Feb 11) *N*=1667
Duration of intervention, (months)	12	12	24
Average monthly rate of enrolment, (patients/month)	0.33	9.5	70
Gender, male, *n* (%)	2 (50)	56 (49)	844 (48.8)
Median age at enrolment (IQR), (months)	7.0 (6.4–7.5)	5.2 (2.8–9.8)	2.5 (1.6–4.8)
Initial PCR tests done, *n* (%)	4 (100)	112 (98.2)	1657 (99.4)
PCR positive, *n*/*N*(%)	0/4 (0)	5/112 (4.5)	97/1657 (5.9)

Over the three time periods, the median age of enrolment of HEI decreased from 7.0 months (MO) to 5.2 months (T-Basic) to 2.5 months (T-PMTCT); *p*<0.001. Over 99% of HEI had at least one PCR done. No positive PCR results were seen during the MO period, with 5 (4.5%) and 97 (5.9%) children testing PCR positive during the T-Basic and T-PMTCT periods, respectively.

### Characteristics of HIC at enrolment

Characteristics of the children who were HIV-infected (HIC) at enrolment are presented in Table 4 [36].

**Table 4:** Characteristics of HIV-infected children (HIC) at enrolment into Tingathe programme

Characteristics	Mentorship Only (Mar 07–Feb 08) *N*=38	Tingathe-Basic (Mar 08–Feb 09) *N*=238	Tingathe-PMTCT (Mar 09–Feb 11) *N*=526
Duration of intervention, months	12	12	24
Average monthly rate of enrolment (patients/month)	3.2	19.8	21.9
Gender, male, *n* (%)	20 (52.6)	116 (48.7)	233 (44.3)
Age, median (IQR) years	2.1 (1–7)	3.9 (1.7–7.6)	5.0 (2.3–8.4)
Received WHO staging, *n* (%) [36]	26 (68.4)	151 (63.4)	315 (59.9)
I	3 (11.5)	53 (35.1)	101 (32.1)
II	4 (15.4)	41 (27.1)	80 (25.4)
III	14 (53.8)	49 (32.5)	118 (37.5)
IV	5 (19.2)	8 (5.3)	16 (5.1)
Missing	12	87	211
Received CD4 testing^a^ , *n* (%)	30 (78.9)	193 (81.1)	347 (66)
Level of Immunosuppression, *n* (%) [36]			
None	12 (40.0)	49 (25.4)	138 (39.8)
Mild	2 (6.7)	34 (17.6)	53 (15.3)
Advanced	5 (16.7)	49 (25.4)	68 (19.6)
Severe	11 (36.7)	61 (31.6)	88 (25.4)

a CD4s measurement provided by MOH, but subject to stock outs. Children with pulmonary tuberculosis no longer needed CD4 measurements for ART eligibility starting in late 2008.

During the MO phase, the median age of enrolment was 2.1 years with 73% of staged patients WHO III or IV and 53.3% of those with a CD4 cell count having advanced or severe immunosuppression. During the T-Basic phase, the median age was 3.9 years with 37.8% of staged patients WHO III or IV and 57.0% of those with a CD4 cell count having advanced or severe immunosuppression. During the T-PMTCT phase, the median age was 5.0 years with 42.6% of staged patients WHO III or IV and 45.0% with a CD4 cell count having advanced or severe immunosuppression. Over the three time periods, the median age of enrolment increased from 2.1 years (MO) to 3.9 years (T-Basic) to 5.0 years (T-PMTCT); *p*=0.016 for the global comparison.

### Total enrolment and mechanism of identification for HIC

By February 2011, a total of 866 HIC were receiving care at the three combined clinics, a 23-fold increase from 2008. Of these, 764 (88.2%) were known to be HIV-infected at enrolment into care. Another 102 (11.8%) were HEI at enrolment, but were later found to be HIV-infected by DNA PCR. Although, as described above, the rate of new enrolment of HIC did not significantly increase from T-Basic to T-PMTCT, when including those enrolled as HEI who later tested PCR positive, the rate of total enrolment did increase from 20.3 per month during T-Basic to 26.0 during T-PMTCT.

**Figure 3:**
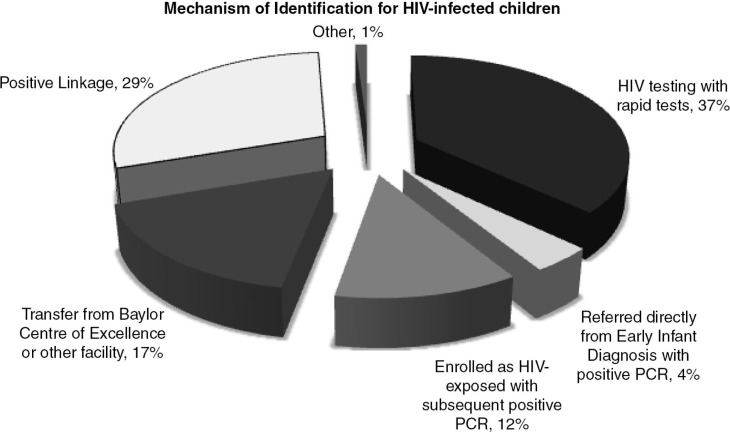
Mechanism of identification for HIV-infected children (HIC). Positive linkage refers to linking children with known HIV-infected status to clinical care services.

Figure 3 depicts the mechanism of identification for the 866 total HIC enrolled into care during the T-Basic and T-PMTCT phases at the three health centres.

HIV testing with rapid tests identified 321 HIC (37%); 102 (12%) were enrolled as HEI but later found to be HIV-infected by PCR; 35 (4%) were referred with a positive PCR test directly from EID services; 146 (17%) were transferred from the Baylor Centre of Excellence or other healthcare facilities; 253 (29%) were identified through positive linkage; and 9 (1%) were identified by other means.

## Discussion

Similar to other countries in the region, identification and enrolment into care of HEI and HIC has been a major challenge in Malawi. Approximately 83,000 HIV-positive pregnant women deliver each year [39] but only 24,682 infants were tested using PCR in 2011 [40]. Roughly 36% of the 180,000 children estimated to be HIV-infected are receiving ART [8,41].

Our work demonstrates that strategies utilizing CHWs can improve identification and enrolment into care of HEI and HIC. We used a multipronged approach combining traditional health-centre-based testing, home-based testing, testing children of adults on ART, positive linkage for patients with known HIV-infected status but not in clinical care and follow-up of mother-infant pairs from PMTCT services. Although several studies have demonstrated the utility of home and community-based testing for adults [18,20,29,30,31,32,34], this is one of the first to describe such an approach with children [42].

Following the addition of CHW support of HIV-positive pregnant women from diagnosis at antenatal clinic, there was a dramatic improvement in HEI enrolment; with a greater than seven-fold increase in average rate of monthly HEI enrolment, and earlier median age of enrolment for the infants. This finding supports results from other studies demonstrating improved follow-up of HEI with integration of PMTCT and EID services [43,44]. Earlier enrolment is critical given the urgency of timely diagnosis of HIV infection as a first step to prompt ART initiation. Indeed, a recent study from South Africa demonstrated that a majority of HIV-infected infants had already progressed to advanced HIV disease by three months of age, suggesting that ART initiation needs to happen even earlier to prevent severe disease progression and increase the odds of starting ART at an optimal health state [45].

Our results also demonstrate the impact of a comprehensive strategy for identification of HIC. Of the 764 HIC newly enrolled into care during the observation period, only 321 were found directly through HIV testing. The remainder either transferred in from other facilities or were children with known HIV status but not in clinical care, found through the process we refer to as positive linkage. These results suggest that although diagnosis and identification of unknown cases is important, it may be equally critical to identify children who have previously been diagnosed but failed to engage in care. The suboptimal linkage of children to clinical care may have been a reflection of a lack of awareness about availability of HIV services for children. Within Malawi, paediatric HIV care lagged behind adult services, with paediatric treatment guidelines first released in 2005 and paediatric ART formulations not available until 2009. Repeat testing may also be an important service, as 15.1% of those testing HIV-positive had previously been tested. For many of these children, guardians were only willing to start their children in care after previous test results had been independently verified. Interestingly, the median age of enrolment increased steadily over the course of the programme from 2.1 years (MO) to 5.0 years (T-PMTCT). This increase likely reflects improved enrolment of HEI, resulting in fewer young children with an HIV-infected diagnosis at enrolment, and more effective PMTCT service delivery resulting in fewer infections overall [23].

The addition of PMTCT activities did not have a significant impact on new enrolment of HIC. This was not surprising, as the case finding strategies for HIC did not change dramatically from T-Basic to T-PMTCT. In fact, case finding likely became more challenging as the easy to find cases had already been enrolled. However, the dramatic increased enrolment of HEI following the addition of PMTCT support services not only increased the number of HEI in care but also the total number of HIC. During the T-PMTCT period, of 1667 HEI enrolled, 97 had a positive PCR. As a result, the rate of total enrolment of HIC increased from 20.2 per month during T-Basic to 26.0 during T-PMTCT. By February 2011, a total of 866 HIC were receiving care at the three combined clinics, a 23-fold increase from 2008.

Our results suggest a significant unmet need for HIV testing of children in high-prevalence settings like Malawi. All of the children within our testing cohort had an indication for testing such as a parent being infected, being an orphan or a specific request from a guardian for testing. With a median age greater than four years at enrolment, the majority should have been screened through the PMTCT and EID programmes. However, the majority were being tested for the first time. These results are similar to those from a survey conducted in Blantyre, Malawi, that found greater than 80% of children of adult patients on ART had not been tested [46].

Our study also demonstrates that while a higher HIV prevalence was seen in health-centre-based testing, the overall yield of the home versus health-centre-based testing strategies was comparable because the absolute number tested through home-based testing was four times higher than through health-centre-based testing. The children identified through the home-based strategy may have been found earlier in their disease course, but further studies are necessary to compare clinical characteristics and outcomes of children identified through these strategies. Both strategies will be important for a comprehensive approach to identification and enrolment of HIC that includes provider-initiated testing and counselling, screening at immunization clinics, and school-based campaigns [20,21]. Our overall testing yield of 4.6% demonstrates that our approach can be effective and an efficient use of limited resources.

There are several limitations to the data in this report. The Tingathe programme utilized multiple concurrent identification strategies and did not track exactly where and how each newly registered patient was identified. For example, in our data collection forms we grouped health centre testing and did not track if the testing was done at tuberculosis clinic, malnutrition clinic or through routine voluntary counselling and testing. Similarly, for positive linkage, we did not track exactly where each patient was found or how they were originally diagnosed. Moving forward, the programme will more closely record the mechanism of identification for all newly enrolled patients, thereby providing opportunities for more detailed analysis. Prospective studies evaluating the yield, cost and clinical outcomes of the individual strategies are also needed. At the time of our study, our programme was one of the few in Malawi that routinely provided longitudinal care for HEI and pre-ART patients. As such, there were no contemporary control groups with which to compare our intervention. The rise in patient enrolment could potentially reflect broader national improvements in EID and paediatric HIV testing. However, several observations support our assertion that the increase in patient enrolment was mainly due to our programmatic activities. First, the number of paediatric patients on ART rose more than 10-fold at the three participating clinics from 2007 to 2011. During this same period, nationally, the number only doubled [37,47]. Furthermore, we have previously reported that at our sites over 80% of all children born to HIV-positive mothers received DNA PCR HIV testing, compared to less than 25% nationally [23,37]. Based on this evidence, we feel the dramatic and immediate increase in patient enrolment following the implementation of our programme strongly supports the efficacy of our approach.

## Conclusions

In summary, this report is one of the first to describe a comprehensive approach to case finding, demonstrating that a multipronged approach utilizing CHWs can dramatically improve identification and enrolment into care of HEI and HIC. This is an area that urgently warrants attention. In the quest for “virtual elimination” of paediatric HIV, much international attention has been focused on the laudable goal of reducing mother-to-child transmission of HIV [48]. However, even if the ambitious UNAIDS goal of reducing the number of new HIV infections among children by 90% is reached [1], tens of thousands of HEI will continue to be infected every year in high-burden countries. As efforts to eliminate new paediatric HIV infections push forward, effective systems to promptly identify and care for HEI and HIC also need to be developed and implemented.
